# Altered visual information processing systems in bipolar disorder: evidence from visual MMN and P3

**DOI:** 10.3389/fnhum.2013.00403

**Published:** 2013-07-26

**Authors:** Toshihiko Maekawa, Satomi Katsuki, Junji Kishimoto, Toshiaki Onitsuka, Katsuya Ogata, Takao Yamasaki, Takefumi Ueno, Shozo Tobimatsu, Shigenobu Kanba

**Affiliations:** ^1^Department of Neuropsychiatry, Faculty of Medical Sciences, Kyushu UniversityFukuoka, Japan; ^2^Departments of Clinical Neurophysiology, Faculty of Medical Sciences, Kyushu UniversityFukuoka, Japan; ^3^Center for Clinical and Translational Research, Kyushu University HospitalFukuoka, Japan

**Keywords:** bipolar disorder, bottom-up, top-down, visual mismatch negativity, visual information processing, windmill pattern, lithium

## Abstract

**Objective:** Mismatch negativity (MMN) and P3 are unique ERP components that provide objective indices of human cognitive functions such as short-term memory and prediction. Bipolar disorder (BD) is an endogenous psychiatric disorder characterized by extreme shifts in mood, energy, and ability to function socially. BD patients usually show cognitive dysfunction, and the goal of this study was to access their altered visual information processing via visual MMN (vMMN) and P3 using windmill pattern stimuli.

**Methods:** Twenty patients with BD and 20 healthy controls matched for age, gender, and handedness participated in this study. Subjects were seated in front of a monitor and listened to a story via earphones. Two types of windmill patterns (standard and deviant) and white circle (target) stimuli were randomly presented on the monitor. All stimuli were presented in random order at 200-ms durations with an 800-ms inter-stimulus interval. Stimuli were presented at 80% (standard), 10% (deviant), and 10% (target) probabilities. The participants were instructed to attend to the story and press a button as soon as possible when the target stimuli were presented. Event-related potentials (ERPs) were recorded throughout the experiment using 128-channel EEG equipment. vMMN was obtained by subtracting standard from deviant stimuli responses, and P3 was evoked from the target stimulus.

**Results:** Mean reaction times for target stimuli in the BD group were significantly higher than those in the control group. Additionally, mean vMMN-amplitudes and peak P3-amplitudes were significantly lower in the BD group than in controls.

**Conclusions:** Abnormal vMMN and P3 in patients indicate a deficit of visual information processing in BD, which is consistent with their increased reaction time to visual target stimuli.

**Significance:** Both bottom-up and top-down visual information processing are likely altered in BD.

## Introduction

Bipolar disorder (BD) is a chronic illness characterized by recurring mood episodes of depression, mania, or mixed states, which often lead to debilitating clinical and functional outcomes. Many patients (30–60%) experience occupational impairment and social dysfunction even during inter-episode euthymic states (Kam et al., 2011). Indeed, a meta-analysis has concluded that BD is characterized by significant deficits in a broad range of cognitive functions that also persist into euthymic phases, including verbal memory, sustained attention, aspects of executive functions, and emotional processing (Andersson et al., 2008). Moreover, BD has been reliably associated with enduring cognitive deficits and abnormal neurophysiological responses such as amplitude- and latency-modulated event-related potentials (ERPs) (Johannesen et al., 2012).

Several auditory ERP components have been found to be impaired in BD (Thaker, 2008), and after much subsequent investigation, are acknowledged as promising potential biomarkers. Mismatch negativity (MMN) is an important auditory ERP that reflects the detection of deviations from an auditory regularity, and is elicited even when attention is not directed to the stimuli. Therefore, MMN is considered to be an index of pre-attentive auditory information processing (Näätänen, 1990). While no abnormalities have been reported in the few studies that have investigated auditory MMN (aMMN) in BD patients (Catts et al., 1995; Umbricht et al., 2003; Salisbury et al., 2007; Hall et al., 2009), the most recent study (Domján et al., 2012) revealed a prolonged pitch-deviant aMMN latency in patients with BD. Although it is increasingly evident that some of these auditory deficits are common in BD, potential visual dysfunction has not yet been sufficiently clarified, in particular, with regard to the pre-attentive (automatic) information processing underlying visual MMN (vMMN). Because individuals with psychiatric disorders often show abnormalities in both auditory and visual information processing (Maekawa et al., 2012), we believe that like aMMN, vMMN is a promising potential biomarker for psychiatric disorders such as BD.

Regarding other ERPs, individuals with BD differ from healthy control subjects in ERP measures of auditory processing elicited by “oddball” discrimination tasks. In these tasks, participants must identify infrequent target tones presented within a series of frequent (standard) tones. Standard tones elicit the P1, N1, and P2 ERP components, whereas target tones additionally elicit the N2 and P3 ERPs. ERP studies of BD have mainly focused on P3, which is a positive-going wave that peaks approximately 300 ms after the presentation of a target tone and is believed to be an index of selective attention and general cognitive efficiency. The peak latency of this component is believed to reflect stimulus-evaluation speed independent of reaction time, whereas its amplitude may represent neural activity underlying attention and memory processes involved in updating stimulus representations (Polich, 2004). Several studies have reported P3 abnormalities in BD patients, the most consistent being increased P3 latency (Muir et al., 1991; Strik et al., 1998; Thaker, 2008; Hall et al., 2009). However, other studies have found no differences on this measure (Salisbury et al., 1998, 1999). Similarly, while several studies have found reduced P3-amplitudes peaks in BD patients (Muir et al., 1991; Salisbury et al., 1998, 1999; O'Donnell et al., 2004; Hall et al., 2009), others have found no amplitude differences (Souza et al., 1995; Strik et al., 1998). Clinically, compared with healthy controls, P3 amplitude was not reduced in a sample of patients suffering from first-episode affective psychosis (primarily BD) (Salisbury et al., 1998). However, reduced P3 amplitude has been reported in BD patients who were in remission for 6 months, suggesting that this measure indexes a relatively stable deficit that remains even after an extended euthymic period (Kaya et al., 2007).

Visual information processing occurs in several stages, with low-level processing occurring up through primary visual cortex (V1), and high-level processing occurring in up-steam visual association areas. It is well known that P1 (the first positive ERP peak after stimulus onset) reflects lower-level visual processing (for a review, Tobimatsu and Celesia, 2006). Studies have shown a reduced P1 in BD patients, suggesting that lower-level visual information processing may be abnormal in BD (Yeap et al., 2009). Alternatively, the reduced P1 may result from deficits in top-down selective attention. Selective attention is the process whereby a subset of input is selected preferentially for further processing, and has two major aspects: bottom-up and top-down. Bottom-up attention is automatically driven by stimuli properties, whereas top-down attention refers to a volitional focusing of attention on a location and/or an object based on current behavioral goals (Ciaramelli et al., 2008). These streams can operate in parallel but bottom-up attention occurs more quickly than top-down attention (e.g., Treisman et al., 1992). Two specific ERP components are candidates for attentional biomarkers, with visual mismatch negativity (vMMN) and visual P3 indicating bottom-up and top-down attention, respectively (Maekawa et al., 2005, 2009). Here, the paradigm settings for standard, deviant, and target stimuli allowed us to test visual information processing systematically, unlike most vMMN studies that have investigated only pre-attentive (automatic) visual information processing. The purpose of the present study was therefore to evaluate bottom-up and top-down visual information-processing systems in BD patients, and to test the relationships between clinical and demographic measurements and vMMN and visual P3 in BD patients.

## Methods

### Participants

Twenty patients with BD (10 females; mean age: 40.8 years; mean education: 14.6 years; time since diagnosis: 11.5 years) and 20 healthy non-medicated control participants (NC; 10 females; mean age: 41.5; mean education: 14.5 years) without a family history of mental illness were recruited. All participants were right handed, between 18 and 60 years of age and had completed grade-school-level education. Exclusion criteria for the participants included a history of a head injury that resulted in loss of consciousness, history of treatment with electroconvulsive therapy, or a history of substance abuse. For control participants, exclusion criteria included a history of substance abuse or a diagnosis of any current or past Axis I psychiatric illness. Groups did not differ significantly from each other in terms of gender, age, or education years. The patients were recruited from Kyushu University Hospital. This study was approved by Research Ethics Committee in Kyushu University Hospital and all participants gave written informed consent. Diagnosis of BD was made using a clinical interview. DSM-IV-TR (American Psychiatry Association, 2000) diagnoses of all patients were confirmed by two experienced psychiatrists. Participants were free of any diagnosed neurological disorders and had normal or corrected-to-normal vision.

The clinical state of patients at the time of testing was assessed using the Structured Interview Guide for Hamilton Depression Rating Scale (SIGH-D) (Williams, 1988) and the Young Mania Rating Scale (YMRS) (Young et al., 1978).

All patients were taking at least one psychotropic medication. To simplify medication status, we focused on mood-stabilizer dosages (lithium and valproic acid).

Participants' demography, clinical measurements, and medication information are summarized in Table 1.

**Table 1:** **Participants' demography, clinical measurements, and medication status information**.

	**BD**	**NC**
N	20	20
Sex (F/M)	10/10	10/10
Age, years	40.8 (11.0)	41.5 (8.7)
Years of education	14.5 (2.1)	14.7 (2.1)
Age at onset	29.7 (10.7)	–
Illness duration, years	12.6 (12.0)	–
YMRS score	1.6 (2.4)	–
SIGH-D score	4.8 (4.8)	–
Lithium dosage (mg)	433.3 (372.7)	–
Number of patients taking lithium	15	–
Valproic acid dosage (mg)	177.8 (311.9)	–
Number of patients taking valproic acid	5	–

Values are expressed as mean (SD).

BD, bipolar disorder; NC, normal control; YMRS, young mania rating scale; SIGH-D, structured interview guide for Hamilton depression rating scale.

### Visual stimuli and procedures

Visual stimuli, apparatus, procedures, and ERP-recording procedures were the same as in our previous studies of healthy adults (Maekawa et al., 2005, 2009) and autism spectrum disorder (Maekawa et al., 2011).

Circular black-white windmill patterns with 90% contrast were presented on a 20-inch CRT monitor and controlled using a ViSaGe graphics board (Cambridge Research Systems Ltd, Rochester, Kent, UK). The visual stimulus subtended 5.8° of visual angle in diameter at a viewing distance of 114 cm. Participants were seated comfortably in a semi-dark room. To divert attention away from the visually deviant stimuli as much as possible, participants were instructed to focus on a story delivered binaurally through earphones while fixing their gaze on the center of the monitor. Moreover, they were instructed to press a button with their right thumb as soon as they recognized a target stimulus on the monitor. Between trial blocks, they were asked to fill out a questionnaire regarding the context of the story that they had heard (story questionnaire).

**Figure 1:**
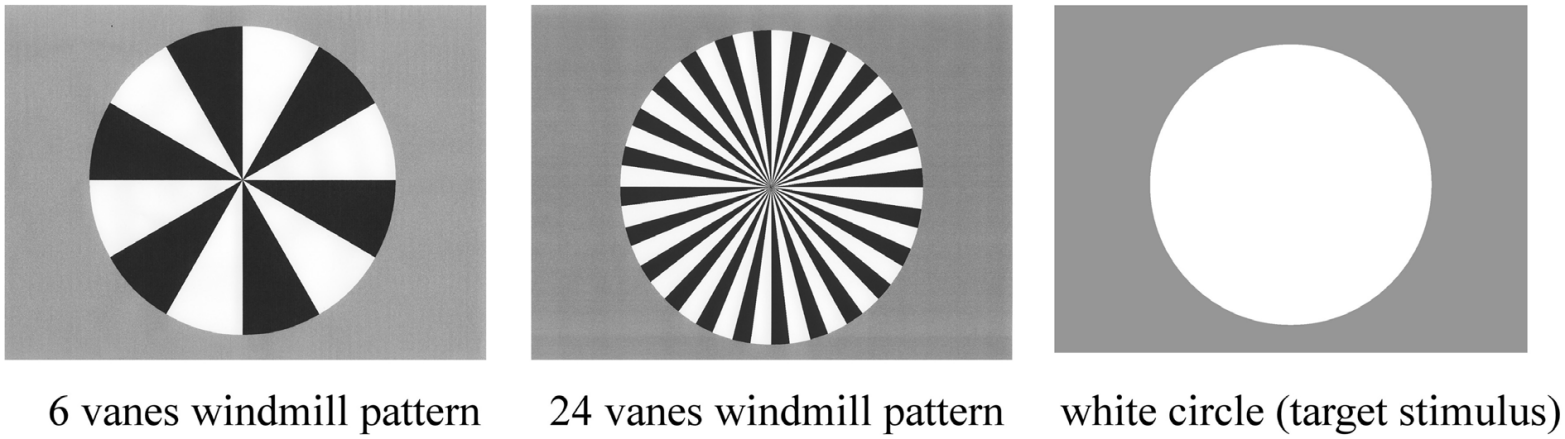
**Three stimulus types used in the present study: six-vane circular black-white windmill pattern stimulus, 24-vane stimulus, and an un-patterned, white circle stimulus.** The two windmill pattern stimuli were adopted as standard or deviant stimuli (counterbalanced across sessions) and the white circle was always used as the target stimulus. Probabilities of standard, deviant, and target stimuli were 8:1:1, respectively.

Standard, deviant, and target stimuli were presented in a random order for 200 ms on the computer monitor (Figure 1). The inter-stimulus interval (ISI) was 800 ms. Stimulus probabilities were 80% (standard), 10% (deviant), and 10% (target).

ERP recordings were composed of two sessions. Standard and deviant stimuli (6-vane and 24-vane windmill patterns) were counterbalanced across sessions, while the target (non-patterned white circle) remained the same throughout the experiment. The target stimulus was the same in the both sessions. The total number of stimuli presented was 1800 (1440 standard, 180 deviant, and 180 target stimuli).

### ERP recordings

EEG was recorded from 128 scalp sites referenced to Cz, using a high-density electroencephalography (EEG) system (Net Station 4.1 Software, Electrical Geodesics, Inc., USA). The impedances of all 128 electrodes were maintained below 50 kΩ. EEG was digitized at 500 Hz and filtered online using a 0.05–200 Hz band-pass filter and stored on a computer.

### Data and statistical analyses

To characterize each subject's degree of attention, the accuracy of answers to the story questionnaire was calculated. Questionnaires consisted of 40 questions, such as “what was the name of the hero?” or “How many persons participated in the operation?” Additionally, reaction time (RT) and accuracy for the target stimuli were also measured as indices of participants' task performance.

EEG data were filtered off-line with a bandpass of 0.05–30 Hz. Digital codes synchronized to the stimulus onset were also stored. At the end of the experiments, EEG epochs associated with each stimulus type were extracted from the continuous record. Epochs with amplitude values exceeding a threshold of ±70 μV were discarded automatically. Artifact-free epochs were then segregated by stimulus code and averaged for each subject. The amplitudes of the ERPs were measured relative to a 100-ms pre-stimulus baseline. The grand average across all subjects in each stimulus condition was also computed. To compare our findings with those of previous studies (Maekawa et al., 2005, 2009, 2011), the average of the two electrodes on either side of the nose (electrodes 126 and 127) was adopted as the reference. Eye movements and blinks were measured from bipolar electrodes above and below the eyes (right, electrodes 14 and 126; left, electrodes 21 and 127). Mean trial numbers for standard, deviant, and target stimuli were 902.7 ± 254.9, 105.1 ± 29.6, and 114.5 ± 34.2 in the BD group and were 908.3 ± 263.4, 115.3 ± 35.3, and 131.8 ± 37.3 in the NC group. There were no significant differences in the number of trials for stimulus type or between subject groups.

Basic ERPs that indicate common neurophysiological information processing were assessed using the P1, N1, and P2 components at the Oz. After overviewing all ERP waveforms, P1, N1, and P2 components from each subject were clearly identifiable (see Table 2). The time windows for P1, N1, and P2 peak amplitudes for standard and deviant stimuli were set at 90 ± 30 ms, 120 ± 40 ms, and 220 ± 50 ms after stimulus onset, respectively. These time windows were considered to include P1, N1, and P2 peaks in all participants (Luck, 2005). Time windows for P1, N1, and P2 peak amplitudes for the target stimuli were set to 80–140 ms, 140–200 ms, and 200–300 ms after stimulus onset, respectively (Luck, 2005). Basic ERP measurements (P1, N1, and P2 peak amplitudes/latencies) at Oz were subjected to a repeated measure analysis of variance (ANOVA) with stimulus type (standard, deviant) as the within-subject variables and participant group (BD, NC) as the between-subject variable. We recognize that measuring basic ERP time-window amplitudes (P1-N1-P2) might be more appropriate than measuring their peak amplitudes (Picton et al., 2000). However, because most previous ERP studies regarding BD have measured peak amplitudes, choosing the same measure makes comparisons between studies more meaningful. Note stimulus type did not include the target stimulus because its pattern was distinct from that of standard and deviant stimuli (i.e., a white circle *vs.* a windmill pattern).

**Table 2:** **Mean peak latencies (ms) and peak amplitudes (μV) of the P1, N1 and P2 at Oz in BD and NC groups**.

**Stimuli**	**ERP peaks**	**Latency (*SD*)**		**Amplitude (*SD*)**	
		**BD**	**NC**	**BD**	**NC**
Standard	P1	82.7 (15.3)^*^	93.0 (12.6)	1.4 (1.7)^*^	4.5 (3.0)
	N1	115.4 (20.3)^*^	135.3 (14.7)	−2.7 (2.1)	−3.2 (4.0)
	P2	223.0 (29.5)	224.0 (23.5)	7.6 (3.3)	8.3 (3.4)
Deviant	P1	73.3 (21.9)^*^	93.8 (12.3)	2.1 (1.5)^*^	5.1 (3.3)
	N1	112.5 (25.9)^*^	135.9 (14.7)	−2.3 (2.3)	−3.7 (3.5)
	P2	218.1 (23.6)	221.9 (22.2)	7.9 (3.5)	7.8 (3.5)
Target	P1	124.0 (20.3)	123.9 (9.8)	4.9 (3.6)	6.7 (3.6)
	N1	167.1 (24.2)	160.7 (10.9)	−1.2 (3.1)	−1.1 (2.8)
	P2	224.6 (41.2)	194.1 (19.0)	2.9 (3.3)	2.1 (2.9)

* P < 0.05.

Values are expressed as mean (SD). BD, bipolar disorder; NC, normal control.

**Figure 2:**
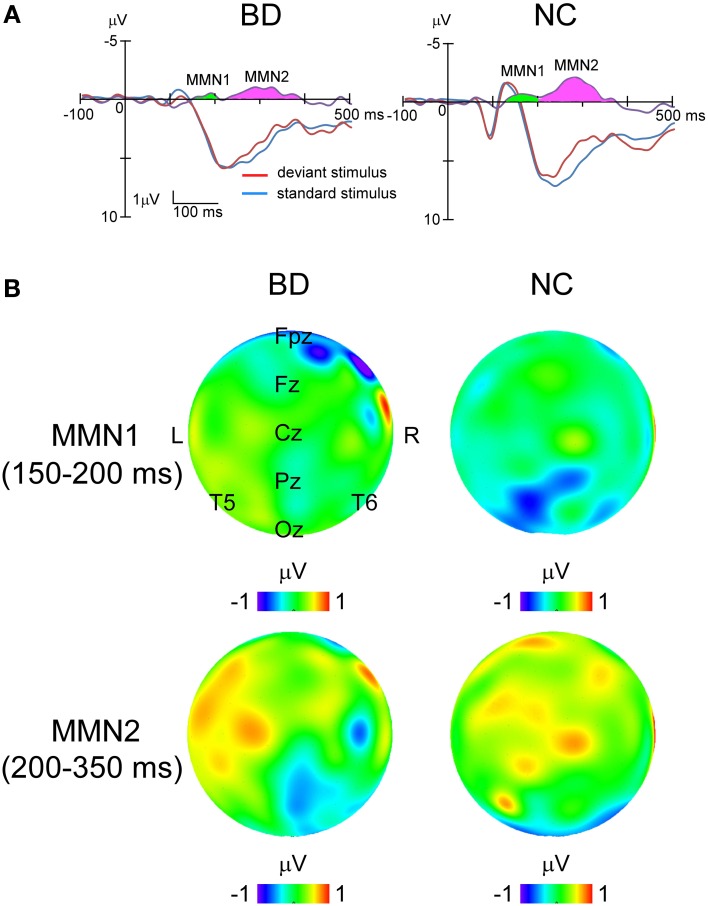
**Grand averaged waveforms to standard and deviant stimuli at Oz and topographical maps of MMNs in each group. (A)** *Blue lines*, standard stimuli. *Red lines*, deviant stimuli. *Purple lines*, their difference waveforms. *Green area*, MMN1. *Pink area*, MMN2. **(B)** Topographical maps of potential in MMN1 and MMN2 for each group. The upper panel shows that MMN1 distributes around Pz area, while the lower panel demonstrates that MMN2 spread over the right occipitotemporal area.

**Figure 3:**
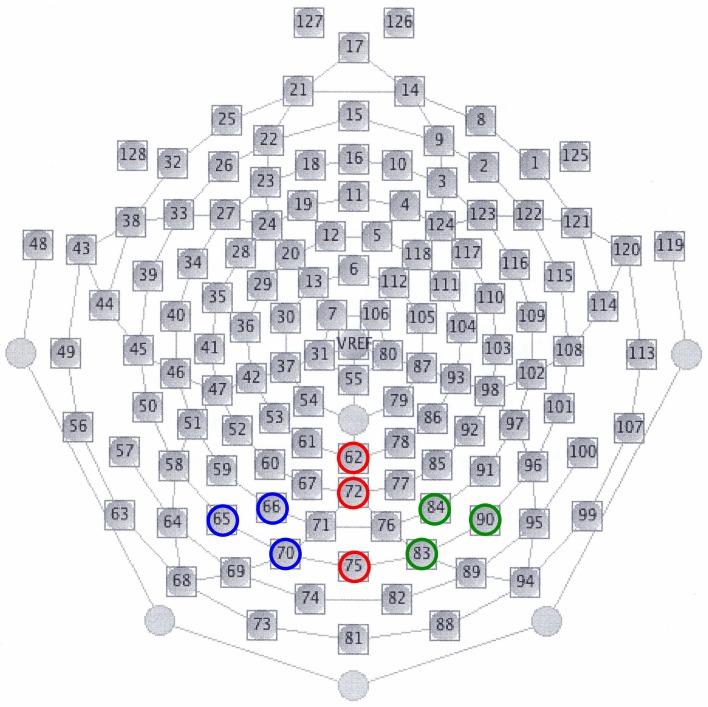
**Selected electrodes of interest for vMMNs.** Electrode numbers 65, 66, and 70 constitute the left occipitotemporal region (blue circles), numbers 62, 72, and 75 the mid-occipitoparietal region (red circles), and numbers 83, 84, and 90 the right occipitotemporal region (green circles). Note that electrodes 62, 75, 58, and 96 correspond to Pz, Oz, T5, and T6 in the international 10–20 system of EEG, respectively.

In all the participants, the response to deviant stimuli at the Oz was more negative than that to standard stimuli during the 150–350 ms following stimulus onset (combined BD and NC groups; paired *t*-test: *t* = 5.186, *P* < 0.001). The time window was justified by visual inspection of grand averaged waveforms and difference waveforms from each participant (see Figure 2), consistent with our previous studies (Maekawa et al., 2005, 2009, 2011). Figure 3 shows the selected electrodes of interest. Electrode numbers 65, 66, and 70 represented the left occipitotemporal region (blue circles), 62, 72, and 75 the mid-occipitoparietal region (red circles), and 83, 84, and 90 the right occipitotemporal region (green circles). Note that Oz corresponds to channel 75 in the EGI net, and this channel was included in the ROI that was later used for statistical analysis. An ANOVA was performed for vMMN mean amplitudes with electrode site (right occipitotemporal, mid-occipitoparietal, left occipitotemporal) being the within subject variable and participant group (BD, NC) the between subject variable. Bonferroni *post-hoc* analysis was performed when significant main effects or interactions were observed.

Attentive visual information processing was evaluated by the N2 and P3 components, which were evoked only in response to the target stimulus. N2 and P3 peak amplitudes/latencies and vMMN mean amplitude were subjected to a repeated measures ANOVA with electrode site (Fz, Cz, Pz, Oz) being the within-subject variable and participant group (BD, NC) the between-subject variable.

## Results

Although behavioral task performance was successfully measured for all participants, data from the two participants in each group were excluded from the ERP analyses because of excessive artifacts in their ERP recordings. Following these exclusions, there were 18 participants in each group. There were no significant differences in sex ratio, age, or education years between the groups.

### Behavioral task performance data

Questionnaire accuracy, response accuracy, and reaction time for target stimuli were evaluated and compared between BD and NC groups using a One-Way ANOVA. There was a marginally significant difference in mean accuracy rates for questions related to the story context [BD, 89.4%; NC, 96.7%; *F*_(1, 19)_ = 3.15, *P* = 0.084], which may indicate either a deficit in attention or short-term memory in BD. Regarding target-stimulus detection, accuracy did not differ between groups [BD, 84.3 ± 2.5%; NC, 89.8 ± 2.5%; *F*_(1, 19)_ = 2.52, *P* = 0.12]. However, compared with the NC group, BD patients showed significantly delayed RTs [BD, 467.4 ± 15.1 ms; NC, 402.4 ± 15.1 ms; *F*_(1, 19)_ = 9.19, *P* = 0.0044].

### Basic ERPs (P1-N1-P2)

**Figure 4:**
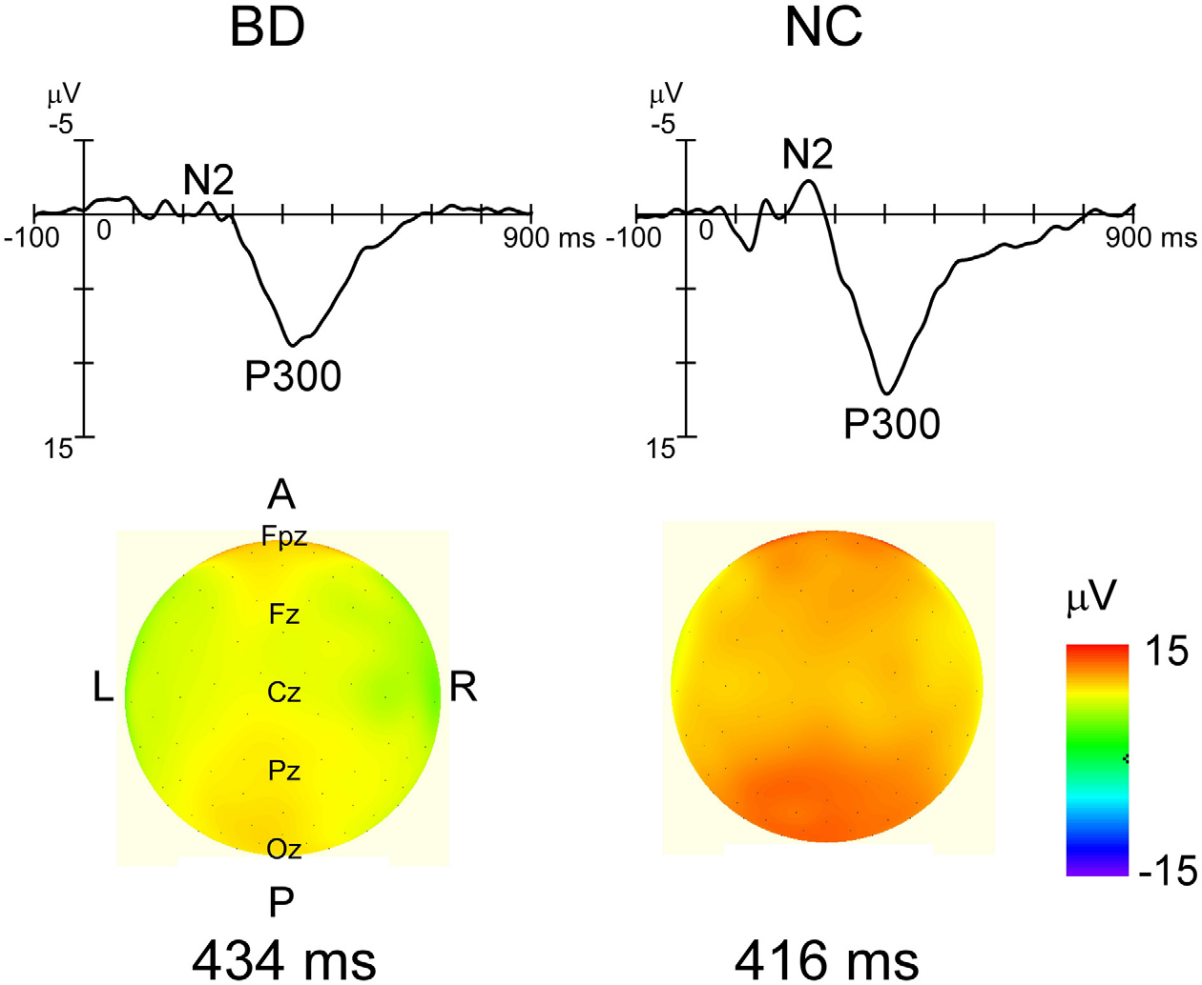
**Grand averaged waveforms in response to target stimulus at Pz and topographical maps of P3 in each group.** Distinct N2 and robust P3 were observed in each group's waveforms. While P3 latency was not significantly different between the two groups, P3 amplitudes were significantly smaller in the BD group (*P* < 0.05). The P3 amplitude gradient for the NC group is steeper than that in the BD group, which roughly corresponds to the statistical differences.

Grand-averaged ERP waveforms in response to the standard and deviant stimuli are shown in Figure 2. Positive (P1)-negative (N1)-positive (P2) deflections were elicited by all three stimulus type and were maximal at Oz. N2-P3 complexes only appeared with the target stimulus, and were maximal at Pz (Figure 4). Mean latency and peak amplitudes for each common ERP component (P1, N1, or P2) are shown in Table 2.

#### P1

A main effect of group was found for both amplitude and latency of the P1 component [amplitude: *F*_(1, 17)_ = 11.63, *P* = 0.002; latency: *F*_(1, 17)_ = 9.01, *P* = 0.005]^1^. P1 latency was significantly shorter and amplitude was significantly smaller in the BD group compared with the NC group. No main effect for stimulus or an interaction between stimulus and group were found.

#### N1

Although there were no main effects or interactions for N1 amplitude, a main effect of group was observed for N1 latency [*F*_(1, 17)_ = 12.08, *P* = 0.001], with latency in the BD group being significantly shorter than that in the NC group. No main effect for stimulus or an interaction between group and stimulus were found.

#### P2

There were no main effects or interactions for either P2 amplitude or latency.

### N2-P3 complex and vMMNs

#### N2

An ANOVA testing magnitude at electrode site (Fz, Cz, Pz, Oz) × participant group (BD, NC) showed a significant main effect of electrode [*F*_(3, 32)_ = 17.59, *P* < 0.000, partial η^2^ = 0.62]. Bonferroni *post-hoc* comparisons showed that amplitudes at Cz and Pz were significantly larger (more negative) than at Fz (Cz: *P* < 0.001, Pz: *P* < 0.001). There were no significant main effects of participant group or any interactions.

Analysis of latency revealed main effects for both electrode [*F*_(3, 32)_ = 10.36, *P* < 0.001, partial η^2^ = 0.49] and participant group [*F*_(1, 34)_ = 18.54, *P* < 0.001, partial η^2^ = 0.35] were found. There were no significant interactions. *Post-hoc* analysis showed that N2 latencies at Pz and Oz were significantly shorter than those at Fz and Cz (Pz: *P* < 0.001 for Fz, *P* = 0.009 for Cz, Oz: *P* < 0.001 for Fz, *P* = 0.008 for Cz).

#### P3

**Figure 5:**
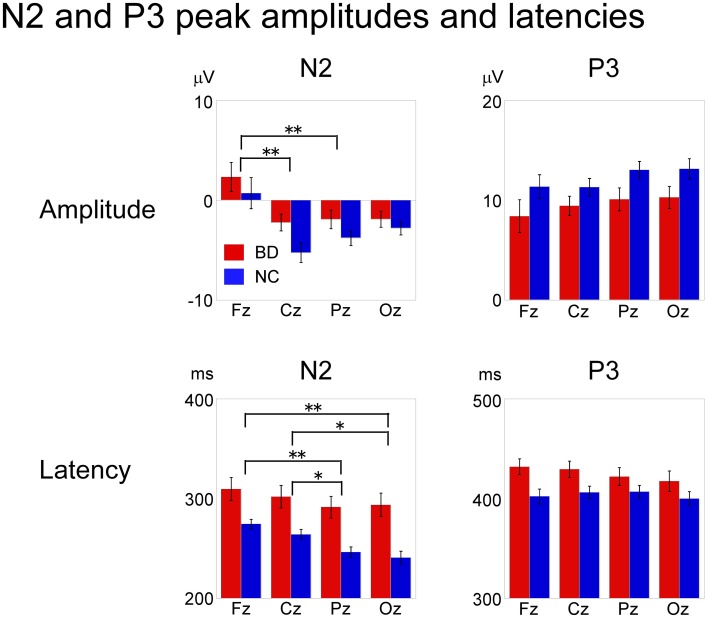
**Mean peak amplitudes and latencies of N2 and P3 at Fz, Cz, Pz, and Oz in BD and NC groups.** N2 latency and P3 amplitude/latency were significantly different between the two groups. ANOVA revealed a significant main effect of electrode site for N2 amplitude and latency, whereas there were no significant statistical differences in P3 amplitude or latency among the electrode sites. Error bar: standard error of mean, ^*^P < 0.01; ^**^P < 0.001.

ANOVAs for both amplitude and latency revealed a significant main effect of participant group [amplitude: *F*_(1, 34)_ = 11.66, *P* = 0.02, partial η^2^ = 0.26, latency: *F*_(1, 34)_ = 4.44, *P* = 0.042, partial η^2^ = 0.12]. There were no significant main effects of electrode site or any interactions (Figure 5).

#### vMMNs

Difference waveforms were constructed by subtracting waveforms generated in response to standard stimuli from those to the deviants. Topographical distributions were inspected to verify that the vMMN occurred around the Oz electrode 150–350 ms after stimulus onset in all participants. Therefore, vMMN amplitude was calculated for each participant as the mean amplitude during that interval. The vMMN consisted of an early peak (MMN1) with a latency between 150 and 200 ms, located predominantly over the parietal area, and a late peak (MMN2) with a latency between 200 and 350 ms located over the temporal area (Figure 2).

While ANOVA revealed no significant main effects or interactions for MMN1 amplitude, significant main effects of both electrode site [*F*_(2, 33)_ = 3.25, *P* = 0.049, partial η^2^ = 0.17] and participant group [*F*_(1, 34)_ = 42.01, *P* < 0.001, partial η^2^ = 0.55] were observed for MMN2. The interaction between electrode site and participant group was also significant [*F*_(2, 33)_ = 3.48, *P* = 0.042, partial η^2^ = 0.18]. *Post-hoc* analysis with multiple comparisons showed that the response at the right occipitotemporal region was significantly larger than that at the mid-occipitoparietal region (*P* = 0.043), but only in the NC group (BD: *P* = 1.000; NC: *P* < 0.001).

### Relationships between ERP components and demographic and clinical measures

**Figure 6:**
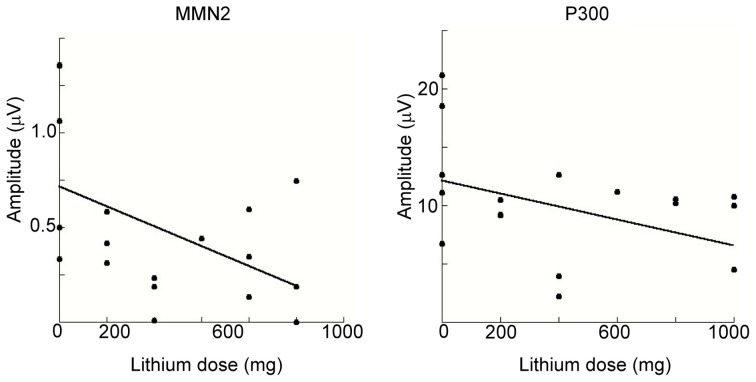
**Spearman's rank correlation analysis for MMN2 (left) and P3 (right) as a function of lithium dosage within the BD patient group.** In the both figures, MMN2 and P3 amplitude are smaller with increasing lithium dosage.

We conducted a multiple regression analysis to examine the relationship between ERP-component amplitudes (MMN2 and P3) and demographic and clinical variables among BD patients. Because mean amplitudes collected from some electrodes might reduce the statistical power, we adopted the amplitude of MMN2 at Oz and that of P3 at Pz for this analysis. The demographic and clinical variables tested with the model were age, sex, years of education, symptom score (YMRS and SIGH-D), mood-stabilizer dosage (lithium or valproic acid), and illness onset age. Figure 6 shows scatterplots of ERP amplitudes and mood-stabilizer dosage. Among BD patients, Spearman's rank correlation analysis revealed that there was a significant relationship between lithium dosage and MMN2 mean amplitude (*R* = 0.48, *P* = 0.043). A significant effect of dosage on P3 amplitude was also observed (*R* = −0.42, *P* = 0.043). Because the number of patients taking valproic acid was small (i.e., five), valproic acid dosage was removed from the correlation analysis. P3 peak amplitude was also significantly correlated with age (*R* = −0.43, *P* = 0.036). Other demographic and clinical variables were not found to be related to MMN2 mean-amplitude or P3 peak-amplitude.

## Discussion

The present study used ERP responses to a non-social visual stimulus to determine whether or not patients with BD show significant differences in visual information processing when compared with healthy individuals. The major differences between BD and control groups are summarized as follows. (1) BD patients performed marginally worse in the auditory context and had significantly slower reaction times. (2) The P1 response to standard and deviant stimuli in BD patients was significantly earlier and smaller than that in the NC group. (3) N1 response latency to standard and deviant stimuli in BD patients was significantly shorter than that in NC group. (4) The N2 latency to the target stimulus in BD patients was significantly delayed and The P3 component was smaller in BD subjects than that in the NC group. (5) MMN2 amplitude in the right occipitotemporal area in BD patients was significantly smaller than that in the NC group. (6) Both MMN2 and P3 amplitudes were significantly correlated with lithium dosage in BD patients. Thus, The ERP profiles for the two groups contained more differences than we expected. Possible explanations for such differences in ERP are discussed below.

### Alteration of early visual processing in BD

We found that compared with controls, the early visual potential (P1) in BD patients was altered, with a significantly shorter latency and smaller amplitude. Most previous studies have not found any significant difference of P1 latency in BD. We presume that other components (such as C1) may overlap the P1 period in the present study. Because the signal to noise ratio can increase because of enormous P1-amplitude reduction, a hidden C1 can emerge that may be mistaken for P1. Even so, the P1-amplitude reduction seen here is consistent with reduction observed in ERP studies of endogenous neuropsychiatric disorders such as schizophrenia (Yeap et al., 2008). Moreover, the reduction seen here was very similar to deficits we reported in patients with schizophrenia using the identical paradigm (Maekawa et al., 2008). This suggests that visual sensory-processing deficits are common to both conditions. Strikingly, these findings are fairly consistent with results from another study in which reduced P1 amplitude to a geometric stimulus (isolated-check image) was demonstrated in BD patients (Yeap et al., 2009). Because the weight of evidence suggests that the P1 deficit is endophenotypic for schizophrenia (Hirano et al., 2010), it will be important for future investigations to establish whether this marker of visual dysfunction indexes shared genetic liability between schizophrenia and BD.

Contrary to our expectation, N1 latency was significantly faster in the BD group than in controls. Whereas ERP studies in BD patients often do not focus on the N1 component, abnormal N1 latency to auditory and/or visual stimuli in BD has been reported (Andersson et al., 2008; Fridberg et al., 2009; Lijffijt et al., 2009). Auditory and visual N1 may share different neurophysiological roles because they are generated from different structures in the brain (supratemporal and extrastriate cortices, respectively). Even so, it is well known that both are modulated by attention (for a review, see Näätänen, 1988). Because the N1 latency and amplitude depend on stimulus conditions (e.g., stimulus type, ISI, intensity, arousal, or attention), sometimes interpreting it in terms of a mechanism for illness is difficult (Rosburg et al., 2008).

### Abnormal attentive processing in BD

The P3, including P3a and P3b, is the most-tested ERP component in patients with BD. Although most studies show abnormal P3 amplitude and/or latency in the grand averaged waveforms in BD patients, whether real statistical differences exist has been controversial (significant: Andersson et al., 2008; Schulze et al., 2008; Fridberg et al., 2009; Hall et al., 2009; Ryu et al., 2010; Jahshan et al., 2012; Johannesen et al., 2012, insignificant: Salisbury et al., 1998, 1999; Bestelmeyer, 2012; Domján et al., 2012). Generally, the group differences and effect size found in ERP measures are not as convincing as the neurophysiological differences, allowing no firm conclusions. However, we found a significantly delayed N2 latency and smaller P3 amplitude. The N2-P3 complex in response to target stimuli is usually called the N2b-complex, and underlies attentional processing for target detection (Näätänen, 1990). The peak-P3 latency is believed to show that stimulus-evaluation speed is independent of reaction time, whereas its amplitude may represent neural activity underlying attention and memory processes involved in updating stimulus representation (Polich, 2004). Despite significantly delayed reaction time, prolonged N2 latency, and reduced P300 amplitude in our BD group, patients followed the contexts of the stories during the examination as well as the control group. Behavioral and neural results indicate that BD patients here likely had a deficit in attention that was obvious behaviorally and neurally, but not clinically.

### Abnormal pre-attentive processing in BD

This is the first report regarding vMMN in patients with BD. Although the existence of a visual analogue of auditory MMN (aMMN) has long been debated, some studies (Pazo-Alvarez et al., 2003; Maekawa et al., 2005, 2009) have demonstrated genuine vMMN that meets the MMN criteria. vMMN is often described as a negativity measured at the occipital electrodes between 150 and 350 ms after the onset of an infrequent (deviant) visual stimulus inserted in a sequence of frequently presented (standard) visual stimuli (Pazo-Alvarez et al., 2003; Czigler, 2007). vMMN is assumed to have similar properties to aMMN, but in the visual modality. Moreover, it can be evoked pre-attentively, which reflects the memory representation of visual-stimulation regularity (Czigler, 2007). Although a number of studies have found converging evidence for the existence of vMMN, there has been little vMMN research related to neuropsychiatric disorders (for a review, see Maekawa et al., 2012). Because there have been few BD reports regarding neurocognitive dysfunction in areas such as sustained attention, selective attention, or visual working memory (Balanzá-Martínez et al., 2008), we hypothesized that vMMN could be a sensitive biomarker for detecting deficits of pre-attentive (automatic) information processing in BD patients. As expected, results in this study demonstrated that vMMN was evoked in the parieto-occipito-temporal area in both subject groups (see topographical maps in Figure 2). Moreover, vMMN comprised an early phase (100–150 ms, MMN1) and a later one (200–350 ms, MMN2), identical to our previous findings (Maekawa et al., 2005), and MMN2 in the BD group was significantly smaller than that in the NC group. Several studies have demonstrated the existence of two vMMN components (e.g., Astikainen and Hietanen, 2009; Kimura et al., 2009) and investigated the sources of vMMN for motion (Pazo-Alzarez et al., 2004; Yucel et al., 2007; Cléy et al., 2013), direction (Kimura et al., 2010), face (Kimura et al., 2012), color (Urakawa et al., 2010a,b; Müller et al., 2012), shape (Kecskés-Kovács et al., 2013), and handedness (Stefanics and Czigler, 2012). While neural activation in the occipital lobe was commonly observed in these studies, several activations in other regions were reported (for instance, posterior parietal cortex, anterior premotor cortex, orbitofrontal cortex, and temporal cortex). Two recent studies (Kecskés-Kovács et al., 2013; Müller et al., 2012) suggest that the earlier component is localized to retinotopically organized regions of the visual cortex and that the later one is generated from the middle occipital gyrus. The early phase has been characterized by deviance-related low-level activation (not allowing for memory issues) while the later one corresponds to the detection of changes based on memory comparisons. Therefore, our vMMN findings, especially MMN2, suggest that patients with BD have limited visual information processing that underlies deficits in pre-attentive memory-based detection of changes in the visual world.

Regarding vMMN laterality between BD and NC groups, MMN2 amplitude in the right occipitotemporal area in the BD group was smaller than that in the NC group. Two reports of vMMN in patients with major depressive disorder (MDD) have been published (Chang et al., 2010; Qiu et al., 2011). Chang et al. (2010) showed that expression-related vMMN at the P8 electrode was smaller in MDD patients compared with normal controls, suggesting a dysfunction in pre-attentive processing of emotional faces. Qiu et al. tested vMMN for duration-deviant stimuli in MDD and found that patients had dysfunctional visual-duration processing in the pre-attentive stage (2011). Although vMMN for short-duration deviants did not differ across groups, vMMN for long-duration deviants was significantly smaller in the right occipitotemporal area of MDD patients. Thus, vMMN is smaller in both MDD and BD patients compared with healthy controls. Although BD and MDD are considered to be different types of illnesses, finding that both conditions are associated with altered vMMN in the right occipitotemporal area implies a common abnormality in visual information.

To date, only two studies have examined the association between vMMN amplitude and behaviorally relevant factors. Stefanics and Czigler (2012) showed that vMMN amplitude to deviant right-hand stimuli correlated with behavioral preference to use the right hand. They concluded that continuously monitoring the identity of the left or right hand is a prerequisite for the ability to automatically transform observed actions into an observer's egocentric spatial reference frame. Gayle et al. (2012) demonstrated that vMMN in individuals with autism-spectrum personality traits were less sensitive to happy emotional expressions, and correlated well with Adult Autism Spectrum Quotient scores. They suggested that vMMN elicited by deviant emotional social expressions may be a useful indicator of affective reactivity and may thus be related to social competency in autism spectrum disorder. These reports indicate that the vMMN is not only an epiphenomenon but also a pre-attentive measurement relevant to behavior.

### Changes in bottom-up and top-down sensory processing in BD

From classical selective-attention capacity-model theory (Kahneman et al., 1992), attention resources in humans are finite and cognitive processing can work successfully only when they are shared correctly. Under a task condition that overloads attention processing, operating efficiency is apparently decreased. A well-known working memory model (Baddeley, 2001), developed from a dual storage model (Atkinson and Shiffrin, 1971), suggests that sensory information (stimulus) automatically enters into sensory registers and is kept as a sensory memory (~500 ms). If selective attention is directed to the sensory memory, intentional processing can work. The sensory register consists of the phonological loop, visuospatial sketchpad, and central executive. The phonological loop and visuospatial sketchpad are controlled and integrated by the central executive system. Therefore, vMMN underlying sensory memory and/or a prediction system (Stefanics et al., 2012a) could reflect bottom-up visual processing (Winkler and Czigler, 2012), while P3 underlying the central executive system could represent top-down visual processing (Saida et al., 2013). According to a more recent model (Friston, 2005), auditory MMN emerges when the incoming stimulus is incongruent with events that are predicted on the basis of learned statistical regularities of the stimulus properties. In line with this, vMMN emerges when a current visual event is incongruent with visual events that are predicted on the basis of extracted sequential rules (i.e., prediction error account of vMMN). Moreover, in this model, forward, backward, and lateral neural connections in the human brain underlie the vMMN. Predictive memory representations of environmental regularities are generated by interactions between multiple levels of a hierarchical system in the brain. Therefore, from these models, our results here can be interpreted as showing abnormal visual working memory systems in BD patients, including both bottom-up and top-down processing.

### Correlation between lithium and ERPs

Correlation analysis revealed significant mutual relationships between age and P3 that were consistent with several previous studies in healthy subjects (Polich, 1991, 2007; Juckel et al., 2012), but are beyond the scope of the current report. Both MMN2 and P3 amplitudes were negatively correlated with lithium dosage (Figure 6) and to the best of our knowledge this is the first report of such correlation.

Several researchers have reported effects of lithium on neuropeptides, cognition, attention, and verbal memory (Bell et al., 2005; Senturk et al., 2007; Nishino et al., 2012; Chiu et al., 2013). For example, brain-derived neurotropic factor (BDNF), which is an important neurotrophin for learning and memory via neurogenesis (Nishino et al., 2012) is itself affected by lithium and the relationship may be explained by the glutamatergic system present in BD patients. In rats, phosphorylation of the NMDA receptor NR2B subunit at Tyr1472 is reduced, suggesting that lithium works to protect against glutamate excitotoxicity in cerebral neurons (Hashimoto et al., 2007). Thus, lithium can affect neurons through its interactions with BDNF, and increasing evidence establishes correlations between BDNF secretion and the glutamate system. In contrast, there is little evidence about its neurophysiological effect on vMMN and P3. One auditory MMN report (Jahshan et al., 2012) showed that there were no significant differences in auditory MMN or P3a between BD patients taking lithium and those that were not. Mood stabilizers such as lithium are given to BD patients to control their affective state, and the effects on attention and cognitive function is therefore very important for their quality of life. More meticulous investigation and prudent interpretations of this issue are therefore needed.

### Methodological considerations

Essentially, vision is always accompanied by attention, which is a basic difference from audition. Therefore, we took scrupulous care of controlling attention in the present experimental design. Participants listened to a story throughout the experiments, and the accuracy of their answers to a questionnaire related to the story was 89.4% and 96.7% in the BD and NC groups, respectively. In addition, accuracy for detecting the correct target was 84.3% and 89.8% in the BD and NC groups, respectively. The high behavioral accuracies assured us that the subjects divided their attention between the auditory task and visual target identification. However, we could not really measure how much attention was diverted toward the deviant stimuli. Even so, the attention specific N2b-P3 complex was activated only by the target stimuli for each condition but not by the deviant stimuli. This result supports the idea that attention was shifted away from the deviant stimulus. Accordingly, we have already tested for attentional leak to the deviant stimulus and concluded our vMMN satisfies the definition of MMN (Maekawa et al., 2005). Moreover, there have been several studies that carefully controlled the direction of attention (e.g., Czigler et al., 2002, 2004; Heslenfeld, 2003; Kimura et al., 2009, 2010; Stefanics et al., 2012a, b) that reported vMMNs similar to the ones we present here. Therefore, even if attention leaked toward the deviant stimulus, we believe that it would not significantly alter our present results.

## Conclusion

Our study is the first to simultaneously investigate both bottom-up and top-down visual information processing in patients with BD. vMMN exhibits properties of early automatic memory-based comparison processing, whereas P3 indexes higher-level, attention-dependent cognitive functions. The deficits in visual information processing that BD patients exhibit seem to be present from the very early stages all the way to higher-level cognitive functions.

### Conflict of interest statement

The authors declare that the research was conducted in the absence of any commercial or financial relationships that could be construed as a potential conflict of interest.

## Footnotes

^1^Analysis of ERP component amplitudes was also carried out on mean amplitudes measured over time windows defined for peak search. The results of this analysis corroborated with that P1 amplitudes for both the standard and deviant stimuli in BD group were significantly smaller than those of NC [*F*_(1, 34)_ = 11.59, *P* = 0.002 and *F*_(1, 34)_ = 9.80, *P* = 0.004, respectively]. One-way ANOVA did not show any significant differences either for N1 or P2 mean amplitude between subject groups.
